# The evidence supporting WHO recommendations on the promotion of hand hygiene: a critique

**DOI:** 10.1186/s13104-018-4012-3

**Published:** 2018-12-17

**Authors:** Patrick Bolton, Timothy J. McCulloch

**Affiliations:** 1 School of Public Health and Community Medicine, University of New South Wales, Randwick, NSW 2031 Australia; 2 Dept Anaesthetics, Royal Prince Alfred Hospital, University of Sydney, Camperdown, NSW 2050 Australia; 3 High St Building, Prince of Wales Hospital, High St, Randwick, NSW 2031 Australia

**Keywords:** Hand hygiene, Evidence based policy, Resource allocation

## Abstract

**Objective:**

To examine the quality of the evidence relied upon by the World Health Organisation (WHO) in promoting hand hygiene with campaigns such as “Save Lives: Clean Your Hands”.

**Results:**

The quality of evidence in the studies quoted by the WHO evidence document is highly variable and the methods used limited. In some of the quoted studies, hand hygiene was the primary outcome, rather than the clinically significant outcome of hospital acquired infection (HAI). When HAI was the primary outcome, it was often poorly defined and reported with scant detail. There was wide variation in the hand hygiene compliance achieved in the intervention studies. The majority of studies where the intervention was a campaign to promote hand hygiene used historical control data with variable attempts to account for the fact that HAI rates may have been declining prior to the hand hygiene intervention. The results from trials with a contemporaneous control were conflicting.

## Introduction

There is little evidence that past quality and safety initiatives have improved health outcomes [1] and there has been a corresponding failure to demonstrate cost-effectiveness [2], despite over 20 years of effort. High quality evidence to inform quality and safety initiatives is often lacking and this may explain some of the difficulty in achieving desired outcomes. When large-scale quality and safety initiatives are implemented on the basis of weak evidence, it becomes likely that scarce healthcare resources will be applied sub-optimally. Furthermore, the goodwill and enthusiasm of healthcare workers for such programs may be compromised.

Over recent years a worldwide effort has been directed to promote hand hygiene in hospitals, with the aim of preventing hospital acquired infection (HAI). Agencies with responsibility for improving the quality and safety of healthcare make strong claims for the benefits of hand hygiene and have prioritised public investment in pursuit of this objective. The World Health Organisation (WHO) has been particularly proactive in promoting hand hygiene with campaigns such as “Save Lives: Clean Your Hands”. Our aim in this article is to examine the strength of evidence relied upon by the WHO for the effectiveness of hand hygiene campaigns.

The oft-repeated assertion, hand hygiene is the single-most effective intervention to reduce hospital acquired infections, is based on several layers of evidence. There is good evidence that microorganisms responsible for HAI can colonise health workers’ hands and that hand hygiene practices can reduce this colonisation. However, it does not necessarily follow that campaigns to promote hand hygiene are an effective intervention. It may be that adequate compliance with hand hygiene cannot be reliably incorporated into routine hospital practice. Also, even if campaigns to promote hand hygiene do lead to a sustained reduction in colonisation of health workers’ hands, parallel transmission of microorganisms by other routes may mean that there is no reduction in rates of HAI.

We are unable to find any published formal systematic review of the effectiveness of hand hygiene for preventing HAI. There is a Cochrane review of compliance with hand hygiene, which found insufficient evidence to conclude that interventions can effectively improve compliance [3], but there is no Cochrane review to address the effect of hand hygiene on the outcome of interest, HAI. An integrative review published in 2008 concluded that the available studies “do not demonstrate a strong relationship between hand hygiene interventions and decreased incidence of healthcare associated infections” [4]. That review found the quality of the available evidence to be poor, with most studies assessed as containing methodological flaws (often fatal) and/or flawed statistical analysis. A similarly conservative conclusion was reached in a 2013 report from the US Agency for Healthcare Research and Quality which noted that hand hygiene is well accepted as a critical patient safety practice but rated the strength of evidence as low [5]. A review commissioned by the UK government concluded there is strong evidence that hand decontamination reduces the carriage of pathogens and “therefore it is logical that the incidence of healthcare related infections is decreased”, but the report did not cite any outcome-based evidence of effectiveness [6].

## Main text

In contrast to the conclusions quoted above, the WHO guidelines on hand hygiene in healthcare advocate the implementation of hand hygiene programs and rate the evidence for hand hygiene before and after touching the patient as “strongly recommended for implementation and supported by some experimental, clinical, or epidemiological studies and a strong theoretical rationale” [7].

Subsequent to promulgation of their guidelines, the WHO published a document entitled, *Evidence of hand hygiene to reduce transmission and infections by multidrug resistant organisms in health-care settings* [8]. The authors of this document reported they had performed an unpublished systematic literature review which identified papers where the key intervention was promotion of hand hygiene and the outcome measure was transmission of multi-drug resistant organisms. They concluded the “great majority of papers offer convincing evidence that improved hand hygiene practices lead to a reduction of HAIs and/or transmission or colonization by multidrug-resistant organisms”. It is reasonable to assume this WHO evidence document includes the strongest evidence available to support the 2009 WHO guidelines. Therefore, we briefly summarise the evidence it cites.

## Limitations of the WHO approach

The WHO evidence document claims to have identified 39 suitable papers. However, it cites just 26 references, only 19 of which are papers with original data relevant to the question. A table in the document summarises eleven studies deemed relevant and higher quality, although no information was provided regarding the criteria used to assess quality.

In our judgement, the quality of evidence in the studies quoted by the WHO evidence document is highly variable. The primary outcome in many of the studies was hand hygiene compliance, with HAI a secondary outcome that was often poorly defined and reported with scant detail. The majority of intervention studies where the intervention was a campaign to promote hand hygiene used historical control data with variable attempts to account for the fact that HAI rates may have been declining prior to the hand hygiene intervention. There was also wide variation in the hand hygiene compliance achieved in the intervention studies.

The majority of the 19 studies quoted by the WHO evidence document that reported original data did not include a contemporaneous control. Of the studies using historical controls, ten studies compared rates of HAI before and after introduction of a hand hygiene initiative in individual wards or hospitals. Changes in rates of HAI were mixed with some studies reporting a decrease (references 9, 18, 20, 23, 24 in the document), some reporting an increase (references 6, 7, 13 in the document), and others reporting little or no change (references 25, 26 in the document). Two studies attempted to analyse the effectiveness of nation-wide hand hygiene initiatives. A time-series analysis from the United Kingdom reported evidence of a positive impact with reduced rates of HAI associated with various stages of a national hand hygiene campaign (reference 17 in the document). The other nation-wide study documented decreasing rates of HAI in Australia after a national hand hygiene initiative and the authors interpreted this as evidence for effectiveness (reference 10 in the document). However, their method for comparing rates of HAI before and after the hand hygiene initiative is open to question and this study could be interpreted as failing to identify any beneficial impact because HAI rates were already falling prior to the intervention (see “Breakout box”).

One group of studies quoted in the WHO evidence document did not directly assess the effectiveness of a hand hygiene intervention. Rather, their approach was to analyse correlations between use of hand hygiene products and rates of HAI. These studies reported decreasing rates of HAI over time, which were positively correlated with increasing usage of hand hygiene products (references 15, 16, 22 in the document). Correlations between rates of HAI and consumption of hygiene products is very low-level evidence for causation.

Only three of the studies quoted in the WHO evidence document included a contemporaneous control and these failed to demonstrate a consistent benefit. One cluster randomised controlled study reported no benefit following a hand hygiene initiative (reference 8 in the document). Another cluster randomised trial conducted across several long-term care facilities (not hospitals) did not report the actual rates of infection but claimed a reduced incidence of infection in the intervention facilities with borderline statistical significance (reference 12 in the document). In the third controlled study, three hospitals introduced a hand hygiene initiative and were prospectively compared to a control hospital (references 14 in the document). There was a decrease in HAI in one intervention hospital but not in the other two, and no information was provided regarding the control hospital.

In summary, the studies quoted in the WHO evidence document provide weak evidence. We do not agree that the references cited in the WHO evidence document justifies the authors’ conclusion that the great majority of papers offer convincing evidence. Given the weak methodology of most of the quoted studies, and their inconsistent results, a contrary hypothesis—that campaigns promoting hand hygiene are ineffective in preventing HAI—is plausible.

Since the promulgation of the WHO campaign, costly efforts have been made to promote hand hygiene. We find it concerning that such a campaign was initiated on what appears to be weak evidence, and it is to be hoped the evidence base can be improved by future studies. It may be difficult and prohibitively expensive to conduct large enough, well designed, prospective trials to address these questions. Hand hygiene is already widely practiced so that a part of any effect may already have been “used up”, potentially reducing the expected effect size, and increasing the necessary sample size, in any future studies. Other difficulties faced by any attempt at a future definitive study include the need to manage intraclass correlation within participating centres. Furthermore, it may be difficult to include a comparison with interventions which seek to reduce the practice of hand hygiene because of its widely perceived benefit. A more feasible approach could be to conduct a robust systematic review of existing material, and consider a meta-analysis if the results of this review make this possible.

Hand hygiene promotion may eventually be found to be an ineffective public health intervention. Public healthcare agencies should lead the synthesis of evidence supporting improvements in clinically important areas. Support for initiatives by public healthcare agencies in the absence of adequate evidence may result in suboptimal allocation of limited health resources and undermine confidence in those agencies. Misdirection of public investment to ineffective interventions may in part account for the observed failure of quality improvement initiatives to deliver improved health outcomes.

## Breakout box

**Fig. 1:**
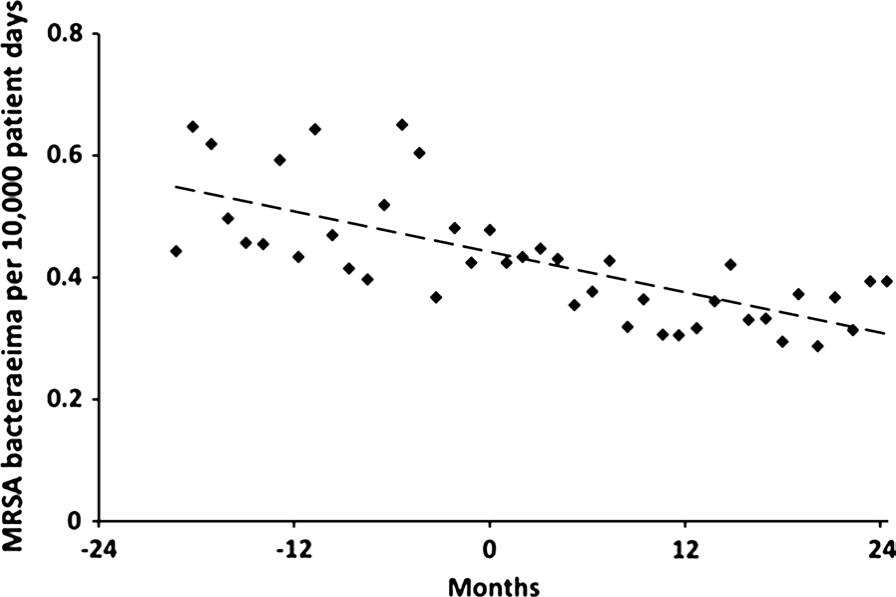
Methicillin-resistant staphylococcus aureus (MRSA) bacteraemia data for Australian hospitals from Grayson et al. [9]. The Australian National Hand Hygiene Initiative was implemented at month-zero. Result of linear regression shown (r = 0.72). Separate regression lines for the data before and after the intervention (not shown) have almost identical slopes

Following the implementation of a nation-wide hand hygiene initiative in Australia, Grayson et al [9] published an analysis of hand hygiene compliance and methicillin-resistant staphylococcus aureus (MRSA) bacteraemia (2011). They analysed data across the 2 years prior to the hand hygiene initiative and the 2 years following the intervention. This study reported a statistically significant downward slope in monthly MRSA bacteraemia rates following the intervention. The authors reported that there was no statistically significant slope prior to the intervention. However, the authors did not formally compare the slopes before and after the intervention. We digitised the MRSA data (from Box 4 of their paper) and could see no evidence of a change in the slope before and after the intervention (Fig. 1). We found the slope was identical before and after the intervention, although the correlation prior to the intervention was weaker due to more scatter (the data prior to the intervention were collected retrospectively).

## Abbreviations

WHO: World Health Organisation
HAI: hospital acquired infection
